# Parasites in pet reptiles

**DOI:** 10.1186/1751-0147-53-33

**Published:** 2011-05-30

**Authors:** Aleksandra Vergles Rataj, Renata Lindtner-Knific, Ksenija Vlahović, Urška Mavri, Alenka Dovč

**Affiliations:** 1 University of Ljubljana, Veterinary Faculty, Institute for Microbiology and Parasitology, Gerbičeva 60, 1000 Ljubljana, Slovenia; 2 University of Ljubljana, Veterinary Faculty, Institute for Health Care of Poultry, Gerbičeva 60, 1000 Ljubljana, Slovenia; 3 University of Zagreb, Faculty of Veterinary Medicine, Department of Biology, Heinzelova 55, 10000 Zagreb, Croatia; 4 Ministry of the Environment and Spatial Planning, Environmental Agency, Vojkova 1B, 1000 Ljubljana, Slovenia

## Abstract

Exotic reptiles originating from the wild can be carriers of many different pathogens and some of them can infect humans. Reptiles imported into Slovenia from 2000 to 2005, specimens of native species taken from the wild and captive bred species were investigated. A total of 949 reptiles (55 snakes, 331 lizards and 563 turtles), belonging to 68 different species, were examined for the presence of endoparasites and ectoparasites. Twelve different groups (Nematoda (5), Trematoda (1), Acanthocephala (1), Pentastomida (1) and Protozoa (4)) of endoparasites were determined in 26 (47.3%) of 55 examined snakes. In snakes two different species of ectoparasites were also found. Among the tested lizards eighteen different groups (Nematoda (8), Cestoda (1), Trematoda (1), Acanthocephala (1), Pentastomida (1) and Protozoa (6)) of endoparasites in 252 (76.1%) of 331 examined animals were found. One Trombiculid ectoparasite was determined. In 563 of examined turtles eight different groups (Nematoda (4), Cestoda (1), Trematoda (1) and Protozoa (2)) of endoparasites were determined in 498 (88.5%) animals. In examined turtles three different species of ectoparasites were seen. The established prevalence of various parasites in reptiles used as pet animals indicates the need for examination on specific pathogens prior to introduction to owners.

## Background

Reptiles have become increasingly common domestic pets. While several reptile species sold as pet animals are bred in captivity, most of them are taken from the wild or are the offspring of wild-caught parents. Wildlife smuggling is on the increase. At the beginning of this century, illegal trade in endangered species had become the third in the world regarding to profit, close behind drugs and arms smuggling. Business may be even more remunerative for other two reasons: culinary specialities and traditional medicine drugs prepared from exotic animals. Further more, non-indigenous species can be found in our environment, upsetting delicate ecosystems eventually leading to the extinction of native species. Reptiles can also be interesting for their potential use in bioterrorism.

Poor capture techniques, compounded by poor or inadequate shipping can kill many reptiles before they reach the pet stores. About 90% of wild-caught reptiles die in the first year of captivity because of physical trauma prior to purchasing or because their owners cannot meet their complex dietary and habitat needs. Reptiles are among the most inhumanely treated animals in the pet trade, because of their special needs for diets and habitats. For many species, the basic requirements for nutrition and housing are unknown, so pet reptiles are highly susceptible to metabolic diseases. In the wild, reptiles rarely come into contact with their own waste or uneaten food, which is a common occurrence in the captivity.

The infestation with parasites plays an important role. Stressful life, concentration of animals and the presence of different species in a small living space actuate development, multiplication and spreading of parasites, which in nature live in cohabitation with their hosts. All these factors suppress the immune response in reptiles and increase the opportunity for viruses, bacteria, yeast and funguses to cause infections and consequent diseases. Reptiles may carry diseases, which can be spread to other animals, other animal species and even to humans [1].

Reptiles can carry viruses e.g. West Nile virus [2], Western Equine Encephalitis [3], bacteria e.g. *Salmonella *sp. [4], *Leptospira *sp. [5], *Chlamydia *sp. [6,7], *Mycobacterium *sp. [7,8], funguses e.g. *Candida *sp., *Trichosporon *sp. [9] or parasites e.g. protozoa - *Cryptosporidium *sp. [10], pentastomes, for example *Armillifer armillatus *[11] and *Porocephalus *sp. [12], ticks like *Amblyomma *sp. and *Hyalomma *sp. [13], mites - *Ophionyssus natricis *[14], which may not make the animal sick but can cause health problems in people. The reptile can be a subclinical carrier of pathogens, for which ticks or other insects are the carriers. Therefore, ticks can play a role in maintaining a rickettsial reservoir (*Borrelia burgdorferi *[15], *Cowdria ruminantium *[16], *Coxiella burnetti*) [13], while mosquitoes can play a role in maintaining the West Nile virus in reptile populations [2]. *Trichinella papuae *and *Trichinella zimbabwensis *are able to complete their entire life-cycle in both poikilothermic (experimentally infected monitors, caimans, pythons and turtles) and homoeothermic animals [17].

The aim of this study was to establish the prevalence of parasites in populations of reptiles, intended to be pet animals in close contact with people. Reptiles imported into Slovenia, specimens of native species taken from the wild, and captive breed species were investigated.

## Materials and methods

A total of 949 reptiles (55 snakes, 331 lizards and 563 turtles), belonging to 68 different species, were examined for the presence of endo and ectoparasites. Among 21 different species of snakes (55 specimens), five species (11 specimens) originated from Slovenia, seven species (23 specimens) were imported from different EU countries and nine species (21 specimens) from Pakistan. Among 32 different species of lizards (331 specimens), eight species (164 specimens) were from Slovenia (163 specimens were from breeding farms and one was from nature), eight species (55 specimens) were imported from different EU countries, eleven species (59 specimens) originated from Pakistan, two species (8 specimens) from the Solomon Islands, two species (three specimens) from the Canary Islands, one species (26 specimens) from Mali, one species (one specimen) from El Salvador and three species (15 specimens) were of unknown origin. Among 13 different species of turtles (563 specimens), eleven species (401 specimens) originated from Slovenian breeding farms, one species (144 specimens) from Lebanon and one species (18 specimens) from Pakistan. Only 17 aquatic turtles belonging to three different species were included in our investigation. Exact reviews of the examined animal species and their origin are presented in Tables 1, 2 and 3.

**Table 1:** The number of examined species of snakes and their origin

SCIENTIFIC NAME	COMMON NAME	NUMBER OF EXAMINED	ORIGIN
*Elaphe guttata*	Corn Snake	10	EU countries*

*Platyceps karelini*	Spotted Desert Racer	6	Pakistan*

*Vipera ammodytes*	Nose-horned Viper	4	Slovenia**

*Lampropeltis triangulum*	Milk Snake	4	EU countries*

*Platyceps ventromaculatus*	Hardwicke's Rat Snake	4	Pakistan*

*Boa constrictor*	Boa Constrictor	3	EU countries*

*Eryx johnii*	Brown Sand Boa	3	Pakistan*

*Spalerosophis atriceps*	Diadem Snake (Black-headed Royal Snake)	3	Pakistan*

*Hierophis gemonensis*	Balkan Racer	2	Slovenia**

*Coronella austriaca*	Smooth snake	2	Slovenia**

*Natrix natrix*	Grass Snake	2	Slovenia**

*Morelia viridis*	Green Python	2	EU countries*

*Python regius*	Ball Python	2	EU countries*

*Zamenis longissimus*	Aesculapean Snake	1	Slovenia**

*Corallus caninus*	Tree Boa	1	EU countries*

*Elaphe obsoleta*	Black Rat Snake	1	EU countries*

*Boiga trigonata*	Indian Gamma Snake	1	Pakistan*

*Gongylophis conicus*	Rough-tailed Sand Boa	1	Pakistan*

*Spalerosophis diadema*	Diadem Snake	1	Pakistan*

Two aquatic species (undetermined species)	/	2	Pakistan*

21 different species		55	

* Snakes were imported from reptile farms.

** Originated from Slovenia - native species.

**Table 2:** The number of examined species of lizards and their origin

SCIENTIFIC NAME	COMMNON NAME	NUMBER OF EXAMINED	ORIGIN
*Uromastyx hardwickii*	Hardwick's Spiny-tailed Lizard	126	Slovenia*

*Iguana iguana*	Green Iguana	25 1	EU countries** El Salvador

*Eublepharis macularius*	Leopard Gecko	25 4	Pakistan EU countries**

*Uromastyx dispar*	Sudan Spiny-tailed Lizard	26	Mali

*Gekko gecko*	Tokay Gecko	15 3	Pakistan EU countries**

*Pogona vitticeps*	Bearded Dragon	15	EU countries**

*Uromastyx aegyptia*	Egyptian Spiny-tailed Lizard	13	Slovenia*

*Physignathus cocincinus*	Chinese Water Dragon	10	unknown****

*Varanus bengalensis*	Bengal Monitor	8	Slovenia*

*Varanus flavescens*	Yellow monitor	7	Slovenia*

*Varanus niloticus*	Nile Monitor	7	Slovenia*

*Corucia zebrata*	Solomon Islands Skink	6	Solomon Islands

*Laudakia melanura*	Black Agama	5	Pakistan

*Teratoscincus scincus*	Common Wonder Gecko	5	Pakistan

*Eublepharis angramainyu*	Iraqi Eyelid Gecko	3	Pakistan

*Chamaeleo calyptratus*	Veiled Chameleon	3 1	EU countries** Slovenia*

*Basiliscus plumifrons*	Green Basilisk	3	EU countries**

*Anolis carolinensis*	Carolina anole	3	unknown****

*Furcifer cephalolepis*	Comoro Islands Chameleon	2	Canary Islands

*Agamura persica*	Persian Spider Gecko	2	unknown****

*Varanus indicus*	Mangrove Monitor	2	Solomon Islands

*Podarcis muralis*	Wall Lizard	1	Slovenia*

*Ophisaurus apodus*	European Glass Lizard	1	Slovenia***

*Anolis equestris*	Knight Anole	1	EU Countries**

*Gekko ulikovskii*	Golden Gecko	1	EU countries**

*Trapelus agilis*	Brilliant Ground Agama	1	Pakistan

*Calotes versicolor*	Oriental Garden Lizard	1	Pakistan

*Phelsuma dubia*	Bright-eyed Day Gecko	1	Canary Islands

*Eutropis macularia*	Bronze Mabuya	1	Pakistan

*Teratoscincus microlepis*	Small-scaled Wonder Gecko	1	Pakistan

*Crossobamon orientalis*	Sind Gecko	1	Pakistan

*Hemidactylus imbricatus*	Carrot-tail Viper Gecko	1	Pakistan

32 different species		331	

* Originated from Slovenia - species from breeding farms

** Lizards were imported from reptile farms.

*** Originated from Slovenia - native species.

**** Originated from animal pet shops in Slovenia.

**Table 3:** The number of examined species of turtles and their origin

SCIENTIFIC NAME	COMMON NAME	NUMBER OF EXAMINED	ORIGIN
*Testudo hermanni*	Hermann's Tortoise	279	Slovenia*

*Testudo graeca*	Spur-thighed Tortoise	144	Lebanon**

*Testudo horsfieldi*	Horsfield's Tortoise	46	Slovenia*

*Geochelone elegans*	Indian Star Tortoise	33	Slovenia*

*Lissemys punctata*	Indian Flapshell Turtle	18	Pakistan*

*Testudo marginata*	Marginated Tortoise	15	Slovenia*

*Trachemys scripta elegans*	Red-eared Slider	10	Slovenia*

*Emys orbicularis*	European Pond Turtle	6	Slovenia*

*Geochelone radiata*	Radiated Tortoise	5	Slovenia*

*Malacochersus tornieri*	Pancake Tortoise	3	Slovenia*

*Geochelone sulcata*	African Spurred Tortoise	2	Slovenia*

*Pyxis arachnoides*	Spider Tortoise	1	Slovenia*

*Testudo kleinmanni*	Egyptian Tortoise	1	Slovenia*

13 different species		563	

* Originated from Slovenia - species from breeding farms - F_1 _and F_2 _generation.

** Turtles were imported from reptile farms.

Anamnestic data, external examination and necropsy of all reptiles have been performed according to Terrell and Stacy [18]. Pathohistological and serological examination and cultivation of pathogens were also performed but are not described in this article. Most of the carcasses were freshly frozen and periodically sent for examination. Not previously frozen dead animals were sent when the reptiles showed clinical signs of diseases prior death. According to our macroscopic findings internal organs, blood, faeces and different swabs were sent for further examination. Digestive tract of all reptiles was systematically examined for the presence of endoparasites. Macroscopically found endo and ectoparasites were examined in our laboratory at the Institute for Microbiology and Parasitology.

For the presence of endoparasites, intestine contents were examined by flotation and sedimentation methods. For flotation saturated NaCl solution with specific gravity of 1.2 was used while sedimentation was performed using tap water. Protozoan parasites were identified by sodium-acetate acetic acid formaldehyde (SAF) method and modified Ziehl-Neelsen staining. In some cases protozoan parasites were identified by native preparation on slides. Identification and determination of endo and ectoparasites was conducted under light microscope.

## Results

### Snakes' parasites

Twelve different species of endoparasites in 26 (47.3%) of 55 examined snakes were determined. In many of them two or more different species of parasites were found. In two cases four different parasitic species were identified: in Ball Python Strongylid eggs, Ascaridae, *Capillaria *sp. and Pentastomida (*Porocephalus crotali*), and in Spotted Desert Racer Strongylid eggs, Acanthocephala, *Cyclospora *sp. and eggs and adults of *Porocephalus crotali*.

At necropsy from two to seven adults of Pentastomida were found on lung surface of Spotted Desert Racer. Local necroses in the lung were present. During necropsy of one *Platyceps karelini *diphtheroid changes in distal part of intestinal tract were detected and at microscopic examination of abrasion of intestinal mucosa *Cyclospora *sp. was seen.

Only fresh carcases of reptiles with previous clinical signs of regurgitation and progressive wasting or with hypertrophic gastritis seen at necropsy changes were tested for the presence of *Cryptosporidium *sp. and those with diarrhoea or diphtheroid lesions of intestinal mucosa were tested for the presence of Trichomonadidae.

For the presence of *Cryptosporidium *sp. in snakes 16 digestive tracts were tested and one Corn Snake was positive. Nine intestines were checked for the presence of Trichomonadidae and one Rough-tailed Sand Boa was positive on *Tetratrichomonas *sp.

Mites from family Macronyssidae (*Ophionyssus natricis*) were present under the scales of one Boa Constrictor and ticks (*Amblyomma *sp.) were found on the Ball Python. Details about species names of endoparasites, their number and percentage and scientific names of snakes are presented in Table 4.

**Table 4:** Number and percentage of positive snakes in regard to infestation with different endoparasites

SCIENTIFIC NAME OF ENDOPARASITES (eggs and/or adults)	NUMBER (%) OF POSITIVE	SCIENTIFIC NAME (NUMBER OF SNAKES)
Strongylid nematoda ^Figure 1^ (*Kalicephalus *sp. and other unidentified species)	11 (20.4)	*Elaphe guttata *(4) *Platyceps karelini *(3) *Zamenis longissimus *(1) *Morelia viridis *(1) *Python regius *(1) *Vipera ammodytes *(1)

Pentastomida ^Figures 2, 3, 4^(*Porocephalus crotali*)	6 (11.1)	*Platyceps karelini *(3) *Platyceps ventromaculatus *(1) *Python regius *(1) undetermined species (1)

Ascarid eggs	4 (7.4)	*Platyceps karelini *(2) *Platyceps ventromaculatus *(1) *Python regius *(1)

*Strongyloides *sp.	3 (5.6)	*Morelia viridis *(1) *Boiga trigonata *(1) undetermined species (1)

*Capillaria *sp.	2 (3.7)	*Coronella austriaca *(1) *Python regius *(1)

Trematod eggs	2 (3.7)	*Hierophis gemonensis *(1) *Platyceps ventromaculatus *(1)

Acanthocephala eggs	2 (3.7)	*Platyceps karelini *(1) undetermined species (1)

*Cryptosporidium *sp. ^Figure 5^	1 (1.9)	*Elaphe guttata *(1)

*Cyclospora *sp. ^Figures 6-and 7^	1 (1.9)	*Platyceps karelini *(1)

*Nyctotherus *sp.	1 (1.9)	*Platyceps karelini *(1)

Oxyurid eggs	1 (1.9)	*Platyceps karelini *(1)

Trichomonadidae (*Tetratrichomonas *sp.)	1 (1.8)	*Eryx johnii *(1)

*Ophionyssus natricis *^Figure 8^	1	*Boa constrictor *(1)

*Amblyomma *sp.	1	*Python regius *(1)

### Lizards' parasites

Eighteen different species of endoparasites in 252 (76.1%) of 331 examined lizards were determined. Two or more different species of parasites were found in many of them. In 38 cases three different species were identified and in two lizards four different species were found. In the Sudan Spiny-tailed Lizard eggs of pinworms (*Pharyngodon *sp.), tapeworms (Anoplocephalidae), *Balantidium *sp., and *Cryptosporidium *sp. were determined. In Tokay Gecko eggs of *Pharingodon *sp., *Physaloptera *sp., *Eimeria *sp. oocysts and Trombiculid mites (*Geckobia *sp.) were found. The presence of *Cryptosporidium *sp. was established in three lizards (two Spiny-tailed Lizards and Leopard Gecko) and Trichomonadidae (*Tetratrichomonas *sp.) in two lizards (Bright-eyed Day Gecko and Chinese Water Dragon). Details about the scientific names of endoparasites, their number and percentage and scientific names of lizards are described in Table 5.

**Table 5:** Number and percentage of positive lizards in regard to infestation with different endoparasites

SCIENTIFIC NAME OF ENDOPARASITES (eggs and/or adults)	NUMBER (%) OF POSITIVE	SCIENTIFIC NAME (NUMBER OF LIZARDS)
Oxyurid eggs ^Figures 9, 10 and 11^ (*Pharyngodon *sp. and other unidentified species)	189 (57.1)	*Uromastyx hardwickii *(89) *Uromastyx dispar *(23) *Iguana iguana *(19) *Eublepharis macularius *(16) *Uromastyx aegyptia *(12) *Physignathus cocincinus *(8) *Pogona vitticeps *(8) *Laudakia melanura *(4) *Agamura persica *(2) *Basilicus plumifrons *(2) *Gekko gecko *(2) *Trapelus agilis *(1) *Corucia zebrata *(1) *Chamaeleo calyptratus *(1) *Teratoscincus scincus *(1)

Strongylid eggs	38 (11.8)	*Uromastyx hardwickii *(27) *Iguana iguana *(3) *Laudakia melanura *(2) *Eublepharis macularius *(2) *Corucia zebrata *(1) *Pogona vitticeps *(1) *Varanus bengalensis *(1) *Varanus niloticus *(1)

*Nyctotherus *sp. ^Figure 12^	33 (10.0)	*Uromastyx hardwickii *(30) *Uromastyx dispar *(3)

Trematod eggs ^Figure 13^	31 (9.4)	*Gekko gecko *(15) *Eublepharis macularius *(7) *Varanus flavescens *(4) *Basilicus plumifrons *(3) *Varanus bengalensis *(1) *Pogona vitticeps *(1)*

Ascarid eggs ^Figures 14 and 16^	23 (6.9)	*Gekko gecko *(15) *Eublepharis macularius *(5) *Varanus niloticus *(2) *Pogona vitticeps *(1)

Pentastomida ^Figure 17^(*Raillietiella *sp.)	23 (6.9)	*Gekko gecko *(15) *Varanus flavescens *(7) *Varanus bengalensis *(1)

*Physaloptera *sp. ^Figures 18, 19 and 20^	21 (6.3)	*Uromastyx hardwickii *(6) *Iguana iguana *(4) *Eublepharis macularius *(3) *Varanus niloticus *(4) *Varanus bengalensis *(3) *Gekko gecko *(1)

Filarioidea ^Figures 21, 22, 23, 24, 25, 26 and-27^ (*Oswaldofilaria *sp. (in monitors) and *Setaria *sp.(in Spiny-tailed Lizards)	18 (5.4)	*Varanus niloticus *(7) *Uromastyx aegyptia *(6) *Varanus bengalensis *(3) *Eublepharis macularius *(1) *Uromastyx dispar *(1)

Cestoda ^Figures 28, 29, 30, 31, 32, 33, 34, 35, 36 and 37^ (*Oochoristica *sp.)	10 (3.0)	*Iguana iguana *(6) *Uromastyx dispar *(4)

Centrorhynchid Acanthocephala ^Figures 38, 39, 40 and 41^	9 (2.7)	*Varanus niloticus *(4) *Uromastyx hardwickii *(2) *Varanus bengalensis *(2) *Laudakia melanura *(1)

*Balantidium *sp.	8 (2.4)	*Iguana iguana *(4) *Uromastyx dispar *(4)

*Cryptosporidium *sp.	3 (0.9)	*Uromastyx dispar *(1) *Uromastyx hardwickii *(1) *Eublepharis macularius *(1)

*Isospora *sp. ^Figure 42^	3 (0.9)	*Pogona vitticeps *(1) *Basilicus plumifrons *(1) *Iguana iguana *(1)

*Eimeria *sp. ^Figure 43^	2 (0.6)	*Phelsuma dubia *(1) *Gekko gecko *(1)

Trichomonadidae (*Tetratrichomonas *sp.)	2 (0.6)	*Phelsuma dubia *(1) *Physignathus cocincinus *(1)

*Capillaria *sp.	1 (0.3)	*Pogona vitticeps *(1)

Heterakidae (*Strongyluris *sp.)	1 (0.3)	*Corucia zebrata *(1)

*Strongyloides *sp.	1 (0.3)	*Varanus bengalensis *(1)

Trombiculid mites ^Figure 44^ (*Geckobia *sp.)	2	*Gekko gecko *(2)

At necropsy from ten to twenty Filarioid nematoda (Onchocercidae, Dirofilariinae, *Oswaldofilaria *sp.) were seen in abdominal cavity under serous membrane and pleura in ten Monitors. In one of the Monitors a mass of round worms (Filarioid nematoda, Spiruroidea, Physalopteridae) was seen in oesophagus and stomach. In seven Spiny-tailed Lizards (*Uromastyx *sp.) Filarioid nematoda (Onchocercidae, Dirofilariinae, *Setaria digitata*) were detected in subcutaneous granulomas at microscopic examination.

Tapeworms (Anoplocephalidae, *Oochoristica *sp.) were seen in intestine of six Green Iguana (*Iguana iguana*) and four Spiny-tailed Lizards (*Uromastyx *sp.) at necropsy. A mass of tapeworms in intestine of Green Iguana has been found. Up to 25 tapeworms were found inside intestines of all four Spiny-tailed Lizards. At necropsy only anaemia was seen.

Up to twenty larvae (cystacanths) of unidentified species belonging to Centrorhynchid Acanthocephala were seen on external side of intestine and mesenterium in six Monitors and two Spiny-tailed Lizards at necropsy.

### Turtles' parasites

Nine different species of endoparasites in 498 (88.5%) of 563 examined turtles were determined. One species of endoparasites was found in 140 turtles, two different species in 182 turtles, while 128 of them had three different species of endoparasites, 36 of them had four, and finally in three turtles five different endoparasites were found. In two Spur-thighed Tortoises, Oxyurid nematoda (Pharyngodonidae, *Tachygonetria *sp.), Ascaridae (*Angusticaecum *sp.) and Strongylid eggs were determined. In one of them *Strongyloides *sp. and eggs of Trematoda and *Balantidium *sp. were found and eggs of Cestoda in the other. In one Marginated Tortoise, Oxyurid nematoda (*Tachygonetria *sp.), Strongylid eggs, *Strongyloides *sp., eggs of Trematoda and *Balantidium *sp. were identified. All the turtles tested for the presence of *Cryptosporidium *sp. and Trichomonadidae were negative.

Ectoparasites (ticks) were found in nine turtles. In one Hermann's Tortoise myasis was confirmed (Calliphoridae, *Lucilia *sp.). Details about the scientific names of endoparasites, their number and percentage and scientific names of turtles are presented in Table 6.

**Table 6:** Number and percentage of positive turtles in regard to infestation with different endoparasites

SCIENTIFIC NAME OF ENDOPARASITES (eggs and/or adults)	NUMBER (%) OF POSITIVE	SCIENTIFIC NAME (NUMBER OF TURTLES)
Oxyurid nematoda ^Figure 45^ (*Tachygonetria *sp.)	459 (81.8)	*Testudo hermanni *(258) *Testudo graeca *(123) *Testudo horsfieldi *(35) *Geochelone elegans *(23) *Testudo marginata *(12) *Geochelone radiata *(4) *Emys orbicularis *(1) *Malacochersus tornieri *(1) *Pyxis arachnoides *(1) *Trachemys scripta elegans *(1)

Strongylid nematoda ^Figure 46^ (*Camallanus *sp. and others, unidentified species)	246 (43.7)	*Testudo hermanni *(128) *Testudo graeca *(77) *Geochelone elegans *(21) *Testudo horsfieldi *(10) *Testudo marginata *(6) *Geochelone radiata *(2) *Trachemys scripta elegans *(1) *Emys orbicularis *(1)

*Balantidium *sp.	147 (26.2)	*Testudo hermanni *(109) *Testudo graeca *(21) *Geochelone elegans *(5) *Testudo marginata *(5) *Testudo horsfieldi *(4) *Malacochersus tornieri *(2) *Testudo kleinmanni *(1)

Ascarid nematoda ^Figures 47, 48 and 49^ (*Angusticaecum *sp.)	114 (20.3)	*Testudo graeca *(82) *Testudo hermanni *(32)

Trematod eggs	47 (8.4)	*Testudo graeca *(38) *Testudo marginata *(9)

*Strongyloides *sp.	21 (3.7)	*Testudo hermanni *(12) *Testudo graeca *(4) *Testudo marginata *(3) *Testudo horsfieldi *(2)

*Nyctotherus *sp.	9 (1.6)	*Geochelone elegans *(5) *Geochelone radiata *(2) *Malacochersus tornieri *(2)

Cestoda eggs	3 (0.5)	*Testudo graeca *(3)

*Amblyomma *sp.	5	*Geochelone elegans *(2) *Geochelone radiata *(2) *Testudo hermanni *(1)

*Hyalomma *sp.	4	*Testudo graeca *(4)

Calliphoridae (*Lucilia *sp.)	1	*Testudo hermanni *(1)

At necropsy of Hermann's Tortoises we frequently (up to 90%) found adult forms of Oxyurid nematodes (*Tachygonetria *sp.) in digestive tract and Ascarid nematodes (*Angusticaecum *sp.) in intestines in approximately 10%.

In Spur-thighed Tortoise we frequently (up to 80%) found Oxyurid nematodes (*Tachygonetria *sp.) in digestive tract and adult forms of Ascarid nematodes (*Angusticaecum *sp.) in intestines in more than 50% of Spur-thighed Tortoise. In the lungs of some juvenile roundworms were also detected.

## Discussion

There is an extremely wide range of different animal species from different parts of the world and a wide range of pathogens, of which some are known and frequently found while others are rare and completely uninvestigated. Their close cohabitation with reptiles demands deep investigation regarding the influence of these species and their microflora on people and autochthonous animal species. The origin of pet reptiles in trade is often unknown; they could be bred in captivity, offspring of wild-caught parents or taken directly from the wild. The variety of different pathogens is very large. The presence of several pathogens in one host and stressful situations can have a negative influence on the health status. Investigation in this field is not satisfactory and many exotic and not familiar pathogens are rarely discovered. There is also a possibility of transmission of the pathogens to people.

A healthy reptile has a number of pathogens, all kept in check by a healthy immune system and the beneficial gut flora. When a reptile is highly stressed or under prolonged moderate to severe stress, the immune system falters. In cases of improper environmental temperatures, starvation, or prolonged dehydration, the beneficial gut flora die off and organisms benign in small numbers gain ascendancy and start causing problems.

### Snakes

**Figure 1:**
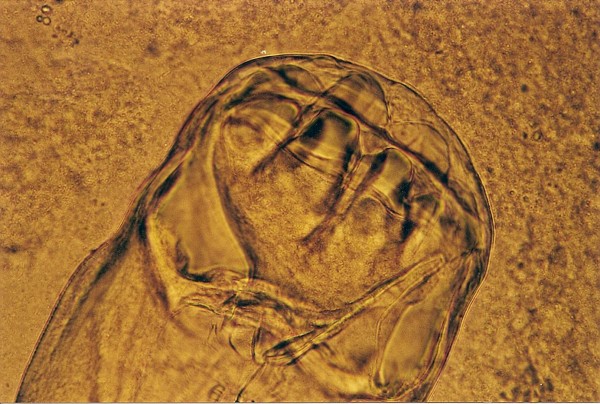
***Kalicephalus *sp. invasion in Corn Snake (*Elaphe guttata*)**.

The most frequent parasites found in snakes (Table 4) were Strongylid nematoda (*Kalicephalus *sp.) in 20.4%. Among eleven snakes (6 different species), four had Strongylid eggs while others had also adults in their intestines. *Kalicephalus *sp. is a hookworm from the order Strongylida, family Diaphanocephalidae. Parasites have a big and deep buccal cavity with milling plates and denticles. The muscularized oesophagus is thick and has bulbous ends (Figure 1). Some morphologic characteristics of *Kalicephalus *sp. are well described by Telford [19].

**Figure 2:**
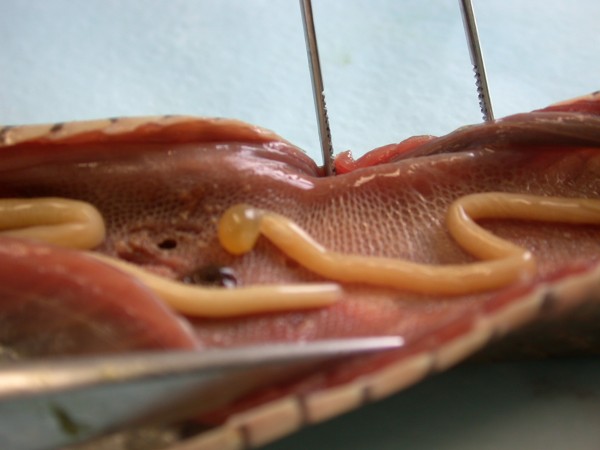
**Pentastomida invasion (*Porocephalus crotali*) in *Platyceps karelini***.

**Figure 3:**
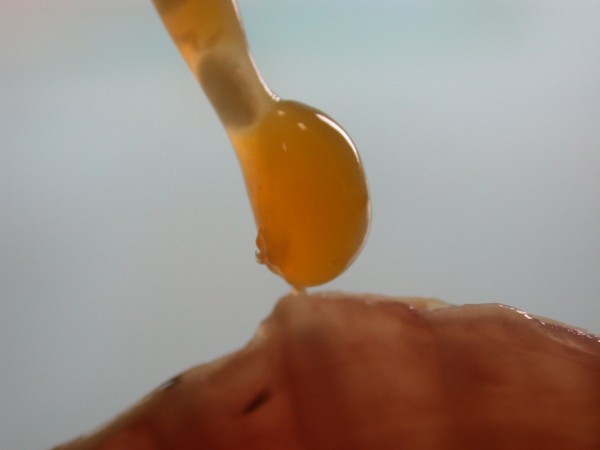
**Pentastomida invasion (*Porocephalus crotali*) (female head) in *Platyceps karelini***.

**Figure 4:**
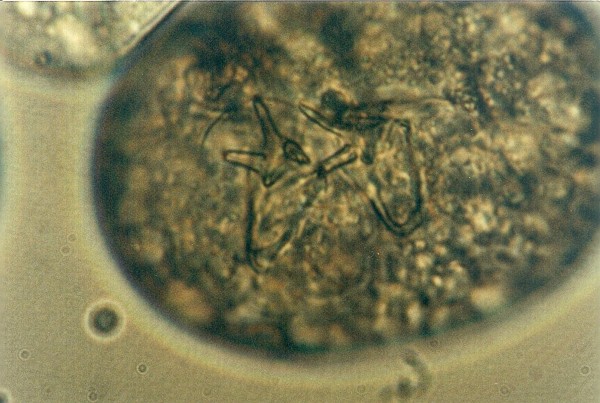
**Pentastomida (*Porocephalus crotali*) embryonated egg**.

Other frequently found parasites were pentastomes. Reptiles periodically eliminate eggs of parasites to the surrounding. Pentastomes do carry zoonotic potential, but among those parasitizing reptiles only *Armillifer *and, more recently, *Porocephalus *(Figure 2) have been unquestionably associated with accidental human infections. However, precautions should always be taken when managing any animals with pentastomiasis. Treatment is very difficult and unsuccessful [20-22]. We detected different forms (four different species) of pentastomes in six snakes (11.1%) and four of them had also eggs in their intestines. The wormlike arthropod *Porocephalus crotali *was found on the surface of the lung. Sexual dimorphism is pronounced, females are larger than males. The head of females is separated from the trunk by a distinct neck (Figure 3). Embryonated eggs had outer and inner shell containing an embryo with clearly visible four legs (Figure 4).

**Figure 5:**
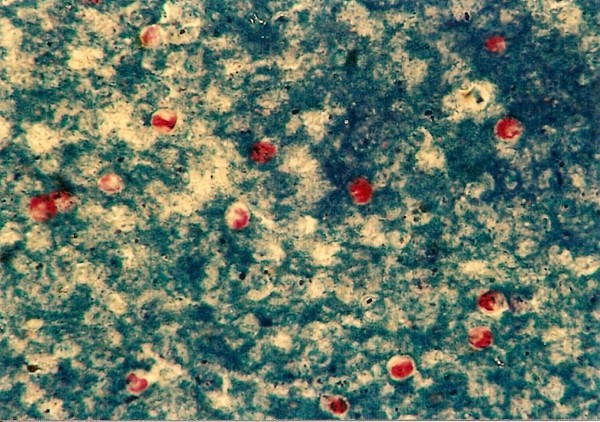
***Cryptosporidium *sp. invasion in intestine of Corn Snake (*Elaphe guttata*)**.

**Figure 6:**
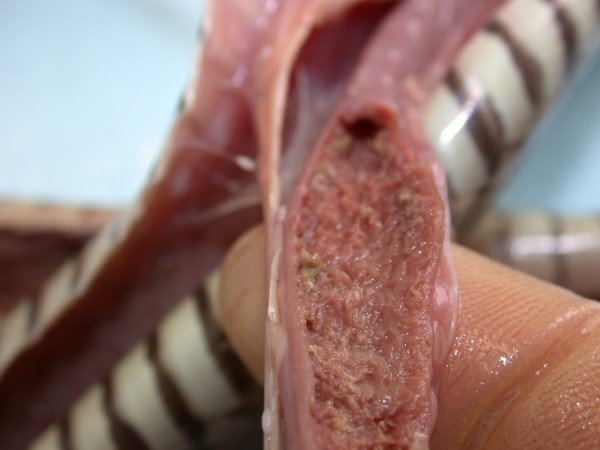
***Cyclospora *sp. invasion in intestine of *Platyceps karelini *- pathoanatomical changes**.

**Figure 7:**
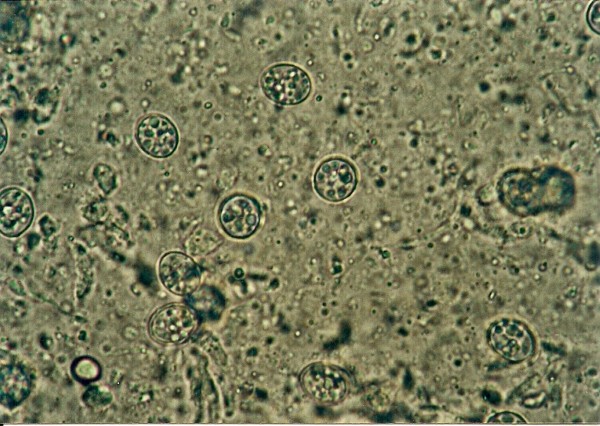
***Cyclospora *sp. invasion in intestine of *Platyceps karelini *- oocysts**.

Ascarid eggs, Oxyurid eggs, *Strongyloides *sp., *Capillaria *sp., Trematoda, Acanthocephala, Trichomonadidae, *Cryptosporidium *sp. (Figure 5), *Cyclospora *sp. (Figures 6 and 7) and *Nyctotherus *sp. were also detected. Similar parasite invasions are described in the literature [21,23,24]. Ascarid eggs which we found were spherical to subspherical with brownish-yellow shell, striated and 6.5 μm thick. We assume the eggs belong to ascaridoid nematoda *Ophidascaris *sp., which is frequently found in pythonid and colubrid snakes. Ascarid nematoda is one of the most important pathogen for snakes and infestation can be fatal [25].

In one Spotted Desert racer pinworm eggs were found. Klingenberg [24] described the same eggs in Ball Pythons. In our case pinworm eggs originated from eaten mice. We also detected some arthropod eggs of mice mites (*Myocoptes musculinus*, *Myobia musculi*) in snake intestines. We agree with Greiner and Schumacher [26] that some not typical eggs in snake faeces can be found because snakes often feed with rodents.

In one Corn Snake a huge dilatation of stomach and diarrhoea was found. Modified Ziehl-Neelsen staining was positive for *Cryptosporidium *sp. This parasite can cause a serious health problem in snakes with hypertrophic gastritis, regurgitation, progressive wasting and death [27]. The latest results indicate the potential zoonotic risk of cryptosporidium isolated from reptiles and not only from mammals [28].

**Figure 8:**
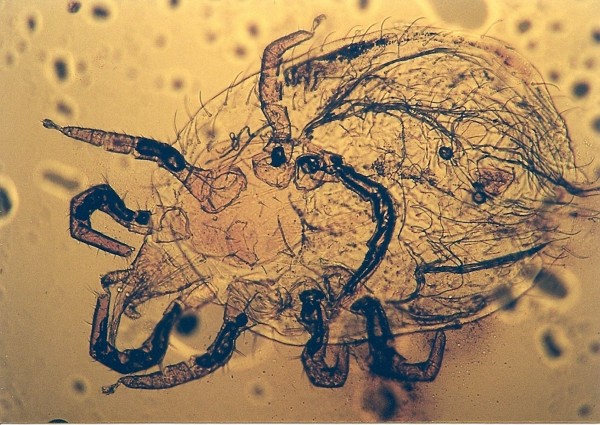
***Ophionyssus natricis *in Red Tail Boa (*Boa constrictor*)**.

The common snake mite, *Ophionyssus natricis *(Figure 8) was found in one Boa constrictor. Schultz described this mite infestation in one pet python and transmission to human [14]. Reptiles suffer from anemia during heavy mite infestation, which can also lead to haemorrhagic septicaemia that is usually fatal. Another author described papulo-vesicular eruptions of the skin in man [19].

In one Ball Python *Amblyomma *sp. ticks were determined.

### Lizards

**Figure 9:**
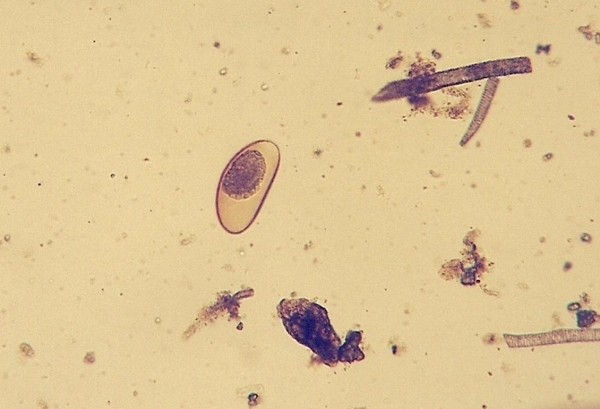
**Pinworm egg (unidentified species) in Green Iguana (*Iguana iguana*)**.

**Figure 10:**
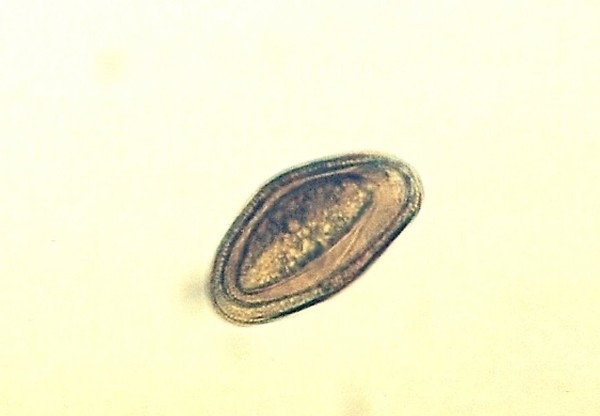
**Pinworm egg (*Pharyngodon *sp.) in Leopard Gecko (*Eublepharis macularius*)**.

The most frequent parasites found in lizards (Table 5) were Oxyurid nematoda in 57.1%. We confirmed these parasites in 15 different species of lizards, most frequently in Chinese Water Dragons (80.0%), Spiny-tailed Lizards (75.2%), Green Iguanas 73.1%, (Figure 9) and Leopard Geckos (55.2%) (Figure 10). Two different shapes of pinworm eggs were seen. One of them was *Pharyngodon *sp. while we could not identify other Oxyurid eggs.

**Figure 11:**
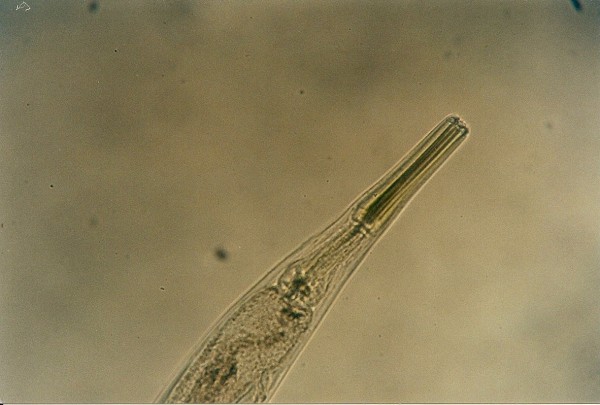
**Anterior end of Pinworm bulbous in Green Iguana (*Iguana iguana*)**.

Pinworms are common in the distal part of the intestine, especially in lizards and turtles. Adults that we found were up to one cm long, white, with characteristic oesophagus with bulbous end (Figure 11). They have a direct life cycle [23]. Lizards living in captivity in small enclosures can re-infect themselves over and over again, which causes the worms to multiply much faster than in the wild.

Klingenberg [23] mentioned that mouse pinworms are also often seen in reptile excrements, but these parasites do not cause diseases in reptiles.

It is important to distinguish between pinworm eggs and eggs of mice mites. In our research the eggs of mice mites were seen more often than pinworm eggs in reptiles eating rodents.

Strongylid nematoda were confirmed in eight different species of lizard (in 11.8%), most frequently in Black Agamas in 40.0% (2/5) and Spiny-tailed Lizards in 21.4% (27/126).

**Figure 12:**
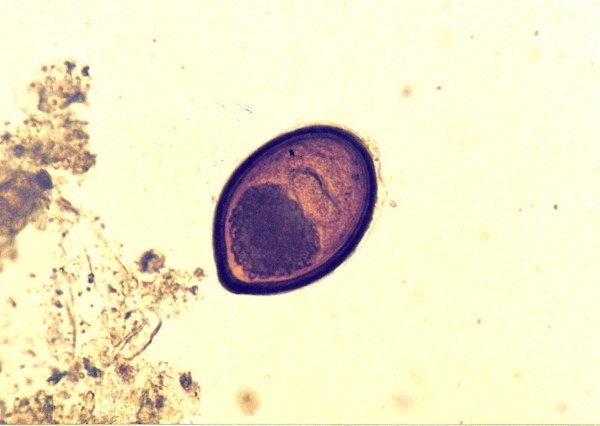
**Ciliophora *Nyctotherus *sp. in Spiny-tailed Lizard (*Uromastyx maliensis*)**.

*Nyctotherus *sp. was determined only in Uromastyx species. Spiny-tailed Lizards (*Uromastyx hardwickii *and *Uromastyx dispar*) were infestated with these ciliated protozoans in 21.7%. Both forms, a bean shaped body with cilia and smaller ovoid cyst with thick membrane and operculum were found. The size of found cysts was 41 × 29 μm (Figure 12). *Balantidium *sp. was determined in lizards (Green Iguana and Spiny-tailed Lizards) in small percentage. Other authors [26,29] described *Nyctotherus *sp. and *Balantidium *sp. as commonly found in herbivorous lizards and also in turtles and snakes with transmission by ingestion of infective cysts. They are not considered as pathogens.

**Figure 13:**
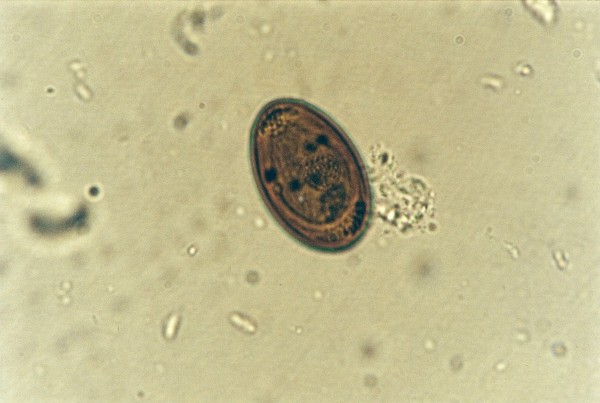
**Miracidium in the egg of Trematoda in Leopard Gecko (*Eublepharis macularius*)**.

Trematoda eggs (Figure 13) were found in 9.4% in six different species, mostly in Tokay Geckos in 83.3%. Digenea trematoda infections occur commonly, particularly in the mouth and oesophagus. Greiner and Schumacher [26] described pulmonary and biliary trematoda. In our case we did not distinguish between these different species. Trematoda can be also found in chameleons but were not established in our research [29].

**Figure 14:**
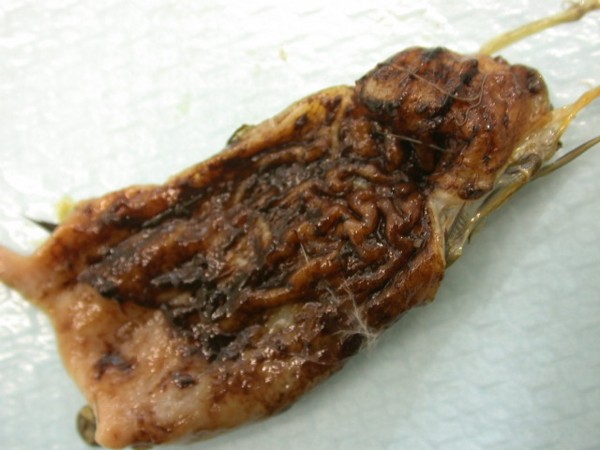
**Invasion with ascarid nematoda in Monitor (*Varanus niloticus*) - stomach**.

**Figure 15:**
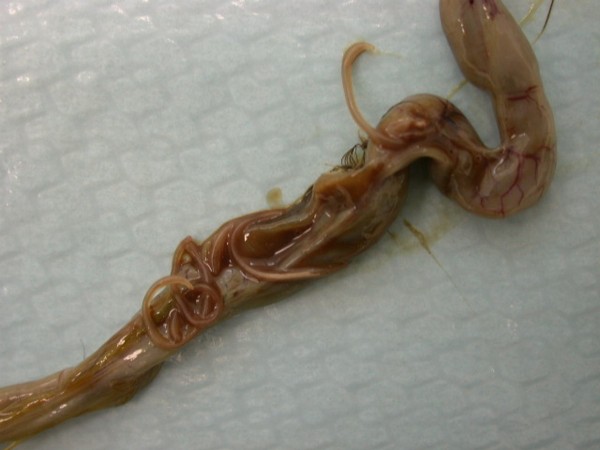
**Invasion with ascarid nematoda in Monitor (*Varanus niloticus*) - intestine**.

**Figure 16:**
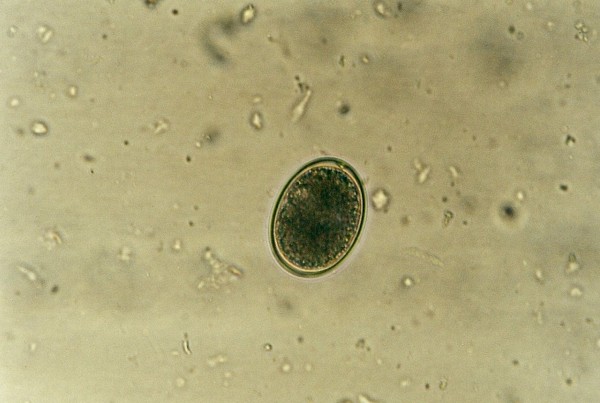
**Ascarid egg in Leopard Gecko (*Eublepharis macularius*)**.

In Figures 14 and 15 invasion with ascarid adults is seen in Monitors at necropsy. Ascarid eggs were found in geckos (Leopard Gecko (Figure 16) and Tokay Gecko) in 46.8% and in one agama (Bearded Dragon). Klingenberg [30] also reported finding roundworms in chameleons imported from Africa.

**Figure 17:**
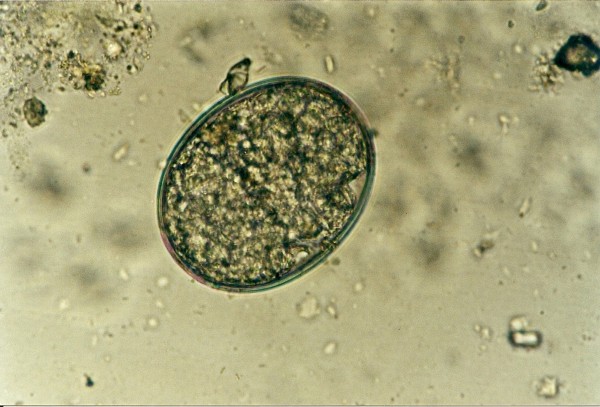
**Pentastomid egg (*Raillietiella *sp.) in Tokay Gecko (*Gekko gecko*)**.

Pentastomid eggs were found in Monitors (Yellow Monitor (100%), Bengal Monitor (12.5%)) and in Tokay Geckos (83.3%). In Tokay Geckos eggs and adults in lungs were confirmed (Figure 17). Klingenberg [31] described Pentastomes also in Bearded Dragons. Parasites found in our research belong to the genus *Porocephalus*, which can pose a potential zoonotic risk. Transmissions of other species of Pentastomes from many different reptiles to people are mentioned and confirmed [12,21,32].

**Figure 18:**
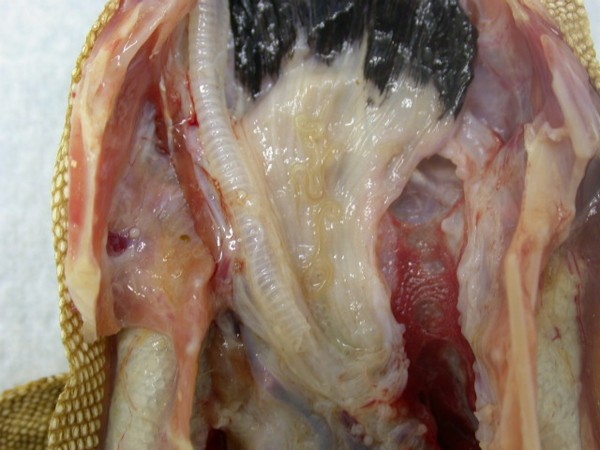
***Physaloptera *sp. invasion in Bengal Monitor (*Varanus bengalensis*)**.

**Figure 19:**
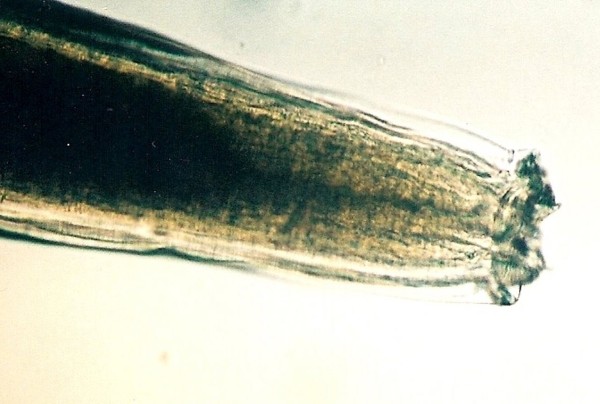
**Anterior end of adult *Physaloptera *sp. in Green Iguana (*Iguana iguana*)**.

**Figure 20:**
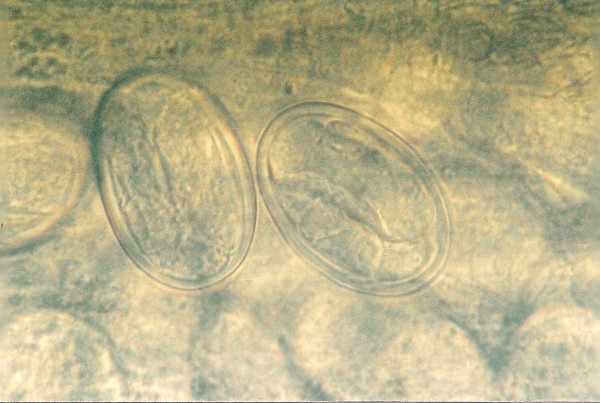
**Eggs in uterus of adult *Physaloptera *sp. in Green Iguana (*Iguana iguana*)**.

*Physaloptera *sp. adults and eggs (Figures 18, 19 and 20) were found in six different species of lizards (Spiny-tailed Lizards, Green Iguanas, Geckos and Monitors) in 6.3%. Telford [19] described massive invasions, particularly in Horned Lizards (*Phrynosoma *sp.).

**Figure 21:**
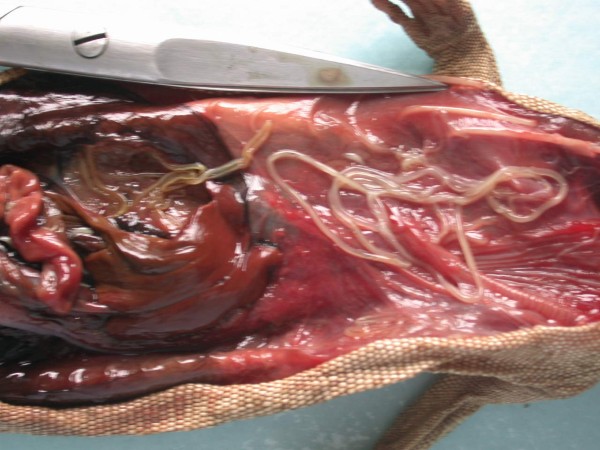
**Filarioidea invasion (Onchocercidae, Dirofilariinae, *Oswaldofilaria *sp.) in Bengal Monitor (*Varanus bengalensis*)**.

**Figure 22:**
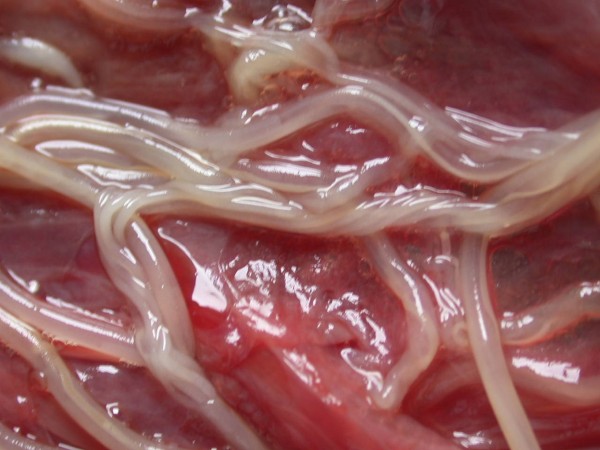
**Filarioidea invasion (Onchocercidae, Dirofilariinae, *Oswaldofilaria *sp.) in Bengal Monitor (*Varanus bengalensis*) - *close view***.

**Figure 23:**
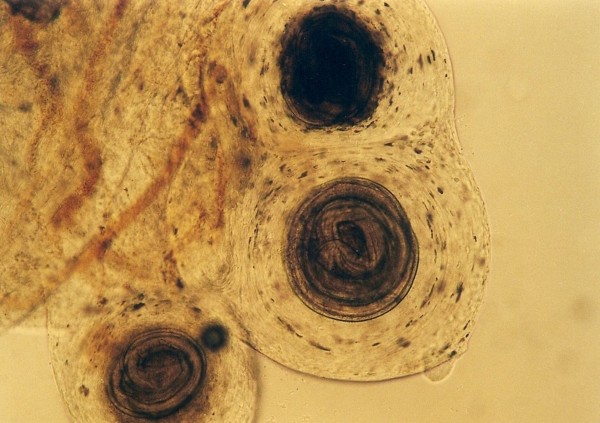
**Nodules of Filarioidea larvae (Onchocercidae, Dirofilariinae, *Setaria digitata*) from peritoneum in Spiny-tailed Lizard (*Uromastyx maliensis*)**.

**Figure 24:**
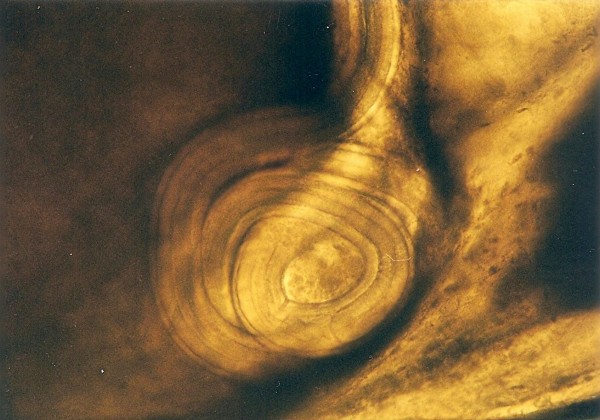
**Nodule of Filarioidea larvae (Onchocercidae, Dirofilariinae, *Setaria digitata*) from peritoneum in Spiny-tailed Lizard (*Uromastyx maliensis*) - close view**.

**Figure 25:**
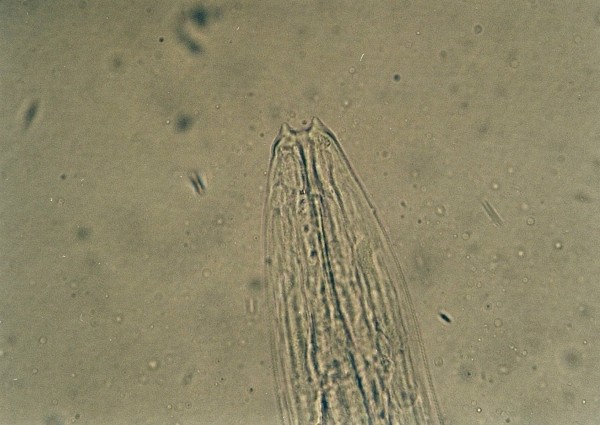
**Anterior end of Filarioidea larva (Onchocercidae, Dirofilariinae, *Setaria digitata*) in Spiny-tailed Lizard (*Uromastyx maliensis*)**.

**Figure 26:**
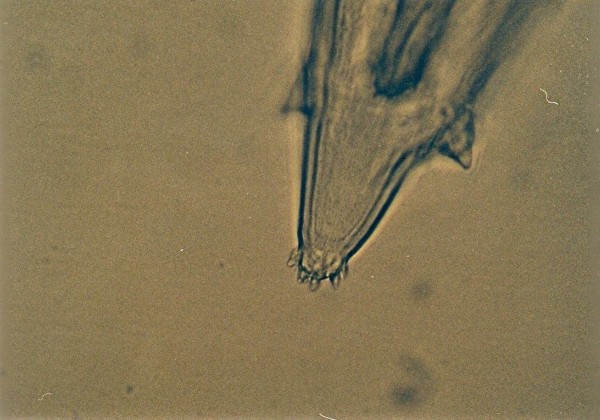
**Posterior end of Filarioidea (Onchocercidae, Dirofilariinae, *Setaria digitata*) female in Spiny-tailed Lizard (*Uromastyx maliensis*)**.

**Figure 27:**
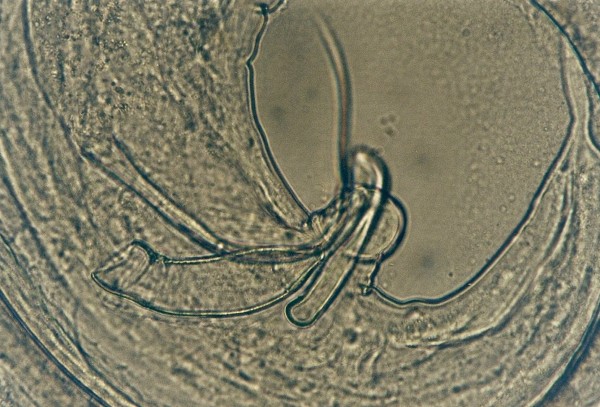
**Spicula of Filarioidea male (Onchocercidae, Dirofilariinae) in Spiny-tailed Lizard (*Uromastyx maliensis*)**.

Filarioid parasites were found in 5.4% of lizards (different species of Monitors, Spiny-tailed Lizards, and in one Leopard Gecko) (Figures 21, 22, 23, 24, 25, 26 and 27). The majority of adult males and females with lot of eggs Onchocercidae, Dirofilariinae, *Oswaldofilaria *sp. were found in the abdominal cavity and nodules on pleura, peritonea and lungs in Monitors.

Nodules were also seen in connective tissue muscles and under the skin of Spiny-Tailed Lizards and in one Leopard Gecko. In those nodules larvae were found. Adult parasites were confirmed in abdominal cavity. Females with specific morphology on the caudal end were seen. Characteristic papillae and tale with papillar comb indicate they belong to family Onchocercidae, Dirofilariinae, *Setaria digitata*.

In our previous research microfilaria were not confirmed in blood smears, but some filarioid worms have been found in the abdominal cavities and subcutaneous granulomas of Monitors [1].

According to literature data these parasites have an indirect life cycle and are usually transmitted by arthropods. Filarioid worms can migrate and cause blisters and ulcers on the skin [25,33].

**Figure 28:**
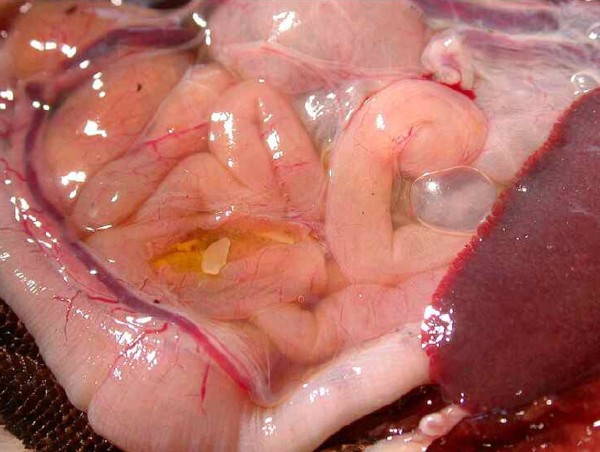
**Cestoda invasion (Anoplocephalidae, *Oochoristica *sp.) in Spiny-tailed Lizard (*Uromastyx maliensis*)**.

**Figure 29:**
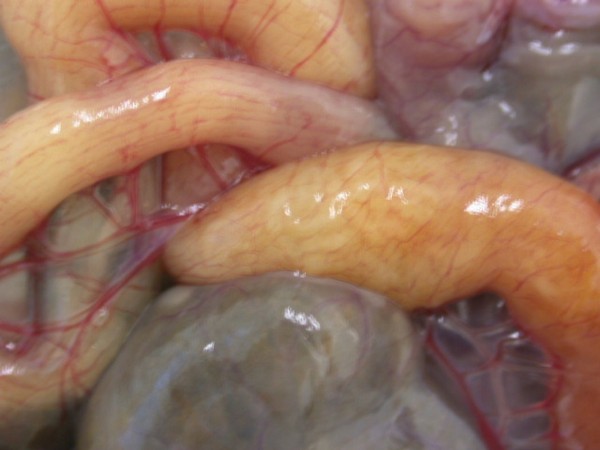
**Cestoda invasion (Anoplocephalidae, *Oochoristica *sp.) in Green Iguana (*Iguana iguana*) - intestines**.

**Figure 30:**
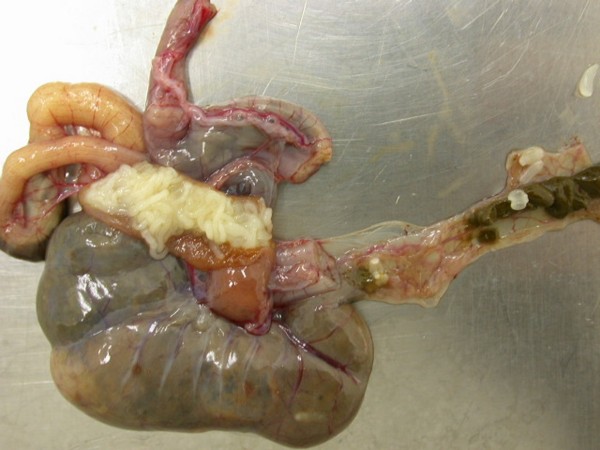
**Cestoda invasion (Anoplocephalidae, *Oochoristica *sp.) in Green Iguana (*Iguana iguana*) - dissected intestines**.

**Figure 31:**
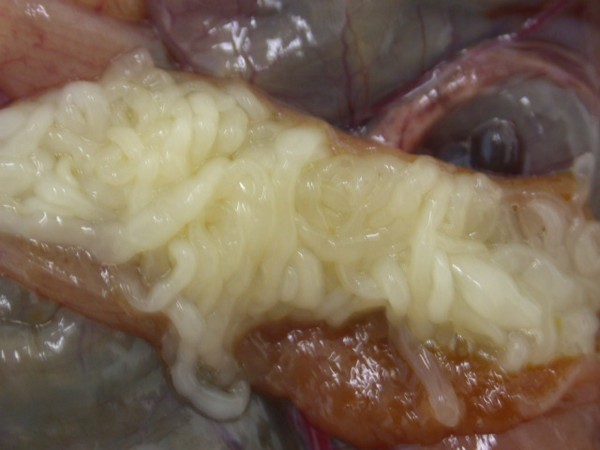
**Cestoda invasion (Anoplocephalidae, *Oochoristica *sp.) in Green Iguana (*Iguana iguana*) - dissected intestines, close view**.

**Figure 32:**
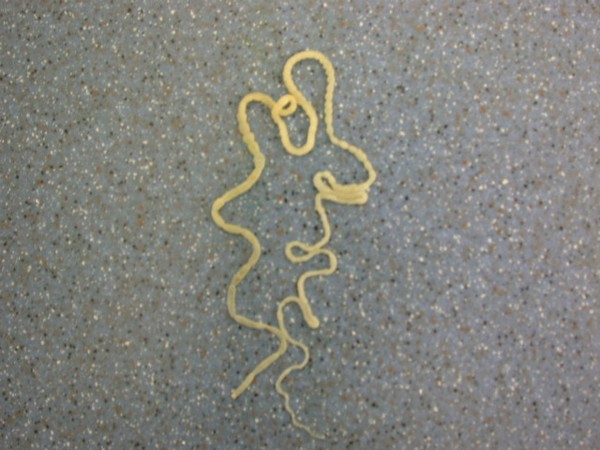
**Cestoda invasion (Anoplocephalidae, *Oochoristica *sp.) in Green Iguana (*Iguana iguana*) - tapeworm**.

**Figure 33:**
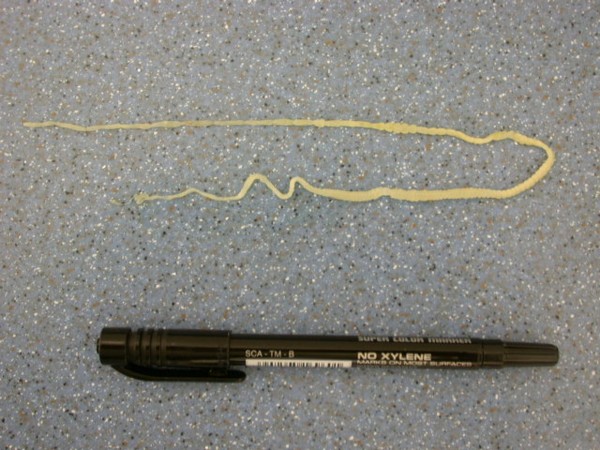
**Cestoda invasion (Anoplocephalidae, *Oochoristica *sp.) in Green Iguana (*Iguana iguana*) - length of tapeworm**.

**Figure 34:**
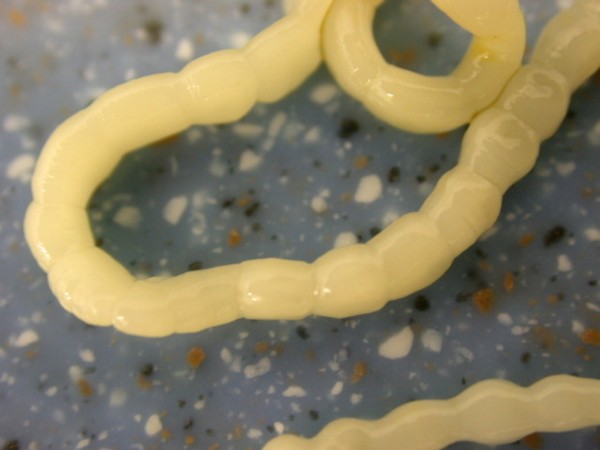
**Cestoda invasion (Anoplocephalidae, *Oochoristica *sp.) in Green Iguana (*Iguana iguana*) - tapeworm, close view**.

**Figure 35:**
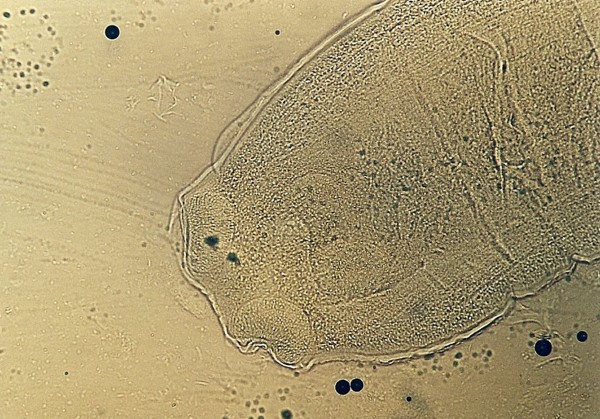
**Anterior end of Cyclophyllidea cestoda (Anoplocephalidae, *Oochoristica *sp.) in Spiny-tailed Lizard (*Uromastyx maliensis*)**.

**Figure 36:**
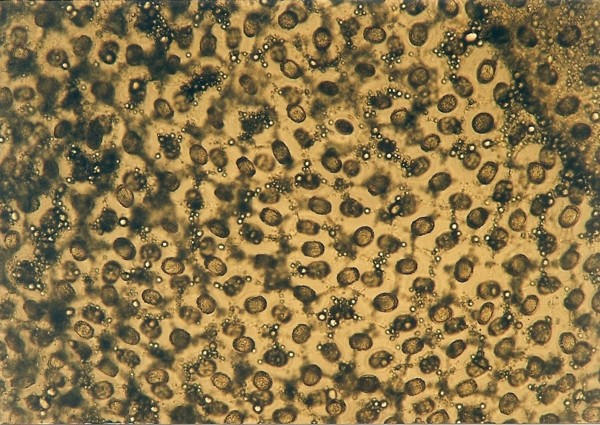
**Eggs in proglottid of Cyclophyllidea cestoda (Anoplocephalidae, *Oochoristica *sp.) in Green Iguana (*Iguana iguana*)**.

**Figure 37:**
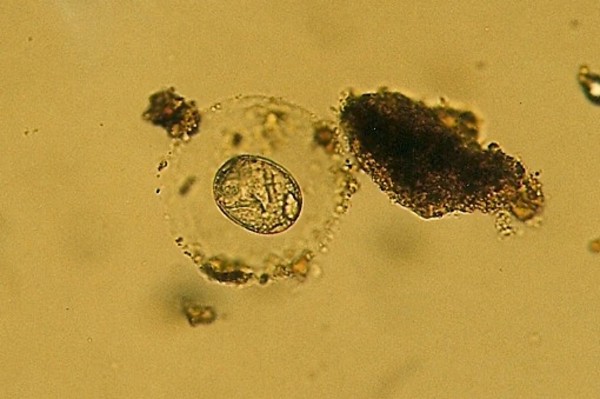
**Hexacanth embryo with membranes of Cyclophyllidea cestoda (Anoplocephalidae, *Oochoristica *sp.) in Green Iguana (*Iguana iguana*)**.

Heavy invasions with Cyclophyllidea cestoda, Anoplocephalidae, *Oochoristica *sp. (Figures 28, 29, 30, 31, 32, 33, 34, 35, 36 and 37) were determined in Green Iguanas and Spiny-tailed Lizards. Two different species of *Oochoristica *sp. were found. One was small, 1.0 to 2.7 cm in length (in Spiny-tailed Lizards) and the other was longer - 28 to 30 cm (Green Iguana). In eggs of both species hexacanth embryos with six hooks with oncospheres in uterine capsule were seen. The specific morphology is described by several authors. McAllister *et al*. described more than 40 species in genus *Oochoristica *in lizards throughout the word [23,31,33,34].

**Figure 38:**
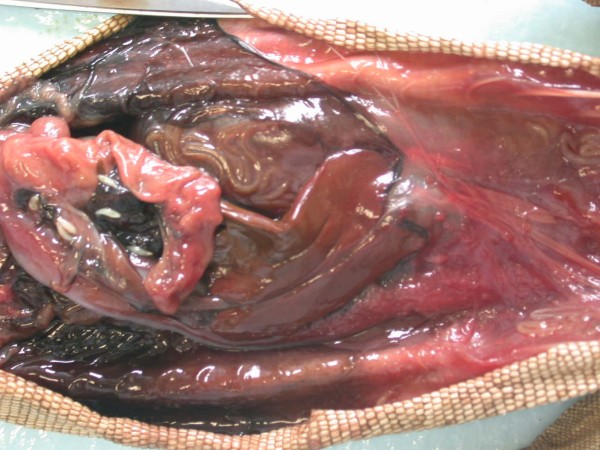
**Acanthocephala and Filarioidea invasion (Onchocercidae, Dirofilariinae, *Oswaldofilaria *sp.) in Bengal Monitor (*Varanus bengalensis*) - pathoanatomical changes**.

**Figure 39:**
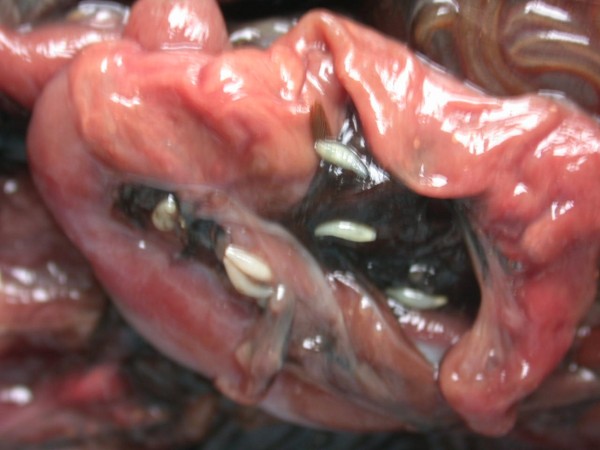
**Acanthocephala and Filarioidea invasion (Onchocercidae, Dirofilariinae, *Oswaldofilaria *sp.) in Bengal Monitor (*Varanus bengalensis*) - close view**.

**Figure 40:**
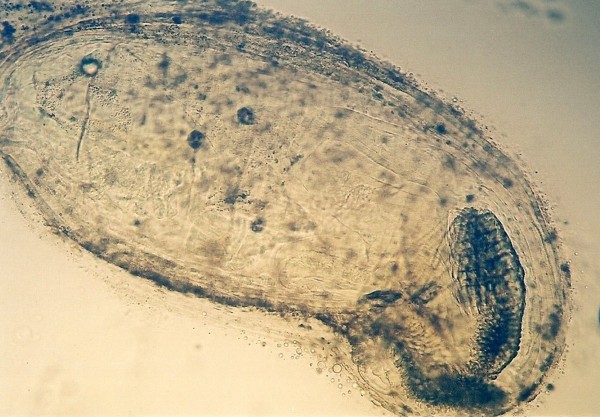
**Centrorhynchid Acanthocephala larva in Nile Monitor (*Varanus niloticus*)**.

**Figure 41:**
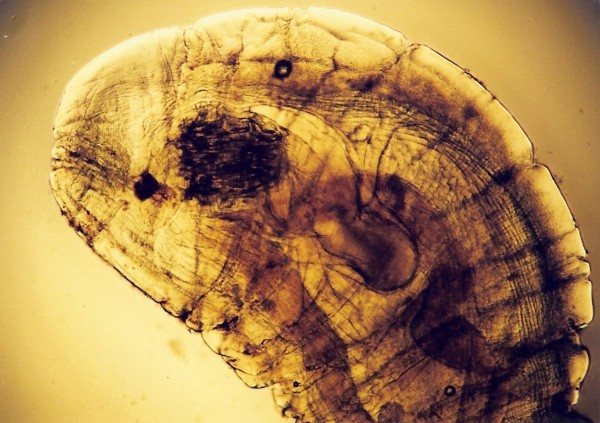
**Anterior end of Centrorhynchid Acanthocephala larva in Spiny-tailed Lizard (*Uromastyx hardwickii*)**.

Acanthocephala larvae (Figures 38, 39, 40 and 41), were about 1 cm long and seen only in the coelomic cavity mostly on intestine serosa. Monitors, Spiny-tailed Lizards and one Black Agama were invaded. We can conclude that these reptiles are paratenic hosts in which larvae are frequently encysted in tissue. We could not find any clinical signs of disease. Similar findings are described by Beck and Pantchev [25].

Cryptosporidiosis in companion and captive exotic animals has received particular attention in recent years due to the public health concerns. Among the exotic animals cryptosporidiosis in snakes and lizards is a chronic life threatening disease [28,35]. Traversa *et al*. [28] described the zoonotic genotype in the faeces of captive European tortoises. In our research we found oocysts in the faeces of clinically healthy Monitors. The source of infection was infected mice, in which oocysts were confirmed and clinical signs were present. That indicates a potential risk for humans.

**Figure 42:**
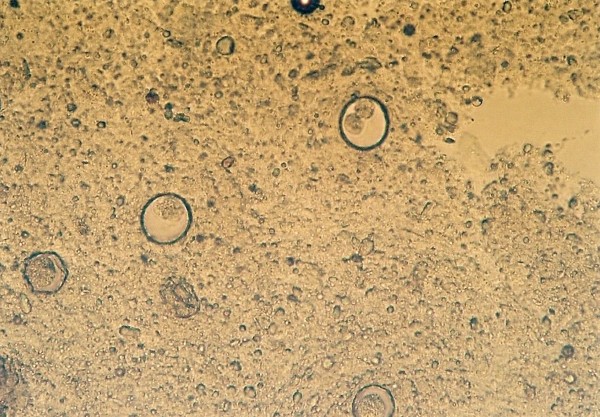
***Isospora *sp. in Bearded Dragon (*Pogona vitticeps*)**.

**Figure 43:**
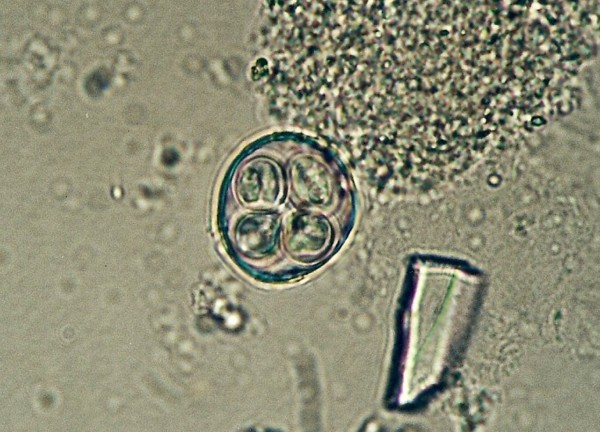
***Eimeria *sp. in Day Gecko (*Phelsuma dubia*)**.

Among Apicomplexa *Isospora *(Figure 42) and *Eimeria *(Figure 43) were determined in low percentage (0.9 and 0.6%). Other authors [1,33] describe these parasites in much higher percentages. Motile protozoans Trichomonadidae were found in two lizards. *Capillaria *sp. eggs, Strongyloides eggs and Heterakidae were detected only in one case.

**Figure 44:**
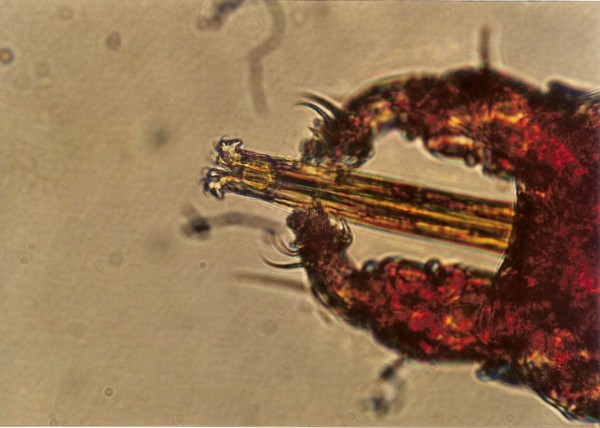
**Chelicerae of Trombiculid mite (*Geckobia *sp.) in Tokay Gecko (*Gekko gecko*)**.

Two geckos had Trombiculid mites (*Geckobia *sp.) around the eyes (Figure 44).

### Turtles

**Figure 45:**
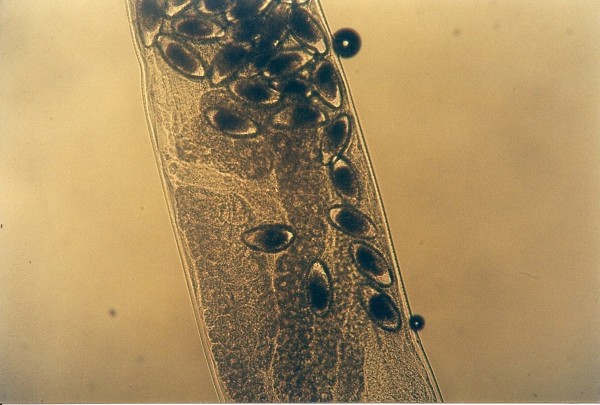
**Oxyurid female with eggs (*Tachygonetria *sp.) in Hermann's Tortoise (*Testudo hermanni*)**.

The most frequent parasites found in turtles (Table 6) were Oxyurid nematoda in 81.8% (Figure 45). We confirmed these parasites in 10 different species of turtles, most frequently in Hermann's tortoises (92.5%). In tortoises we found Oxyurid nematoda (Pharyngodonidae, *Tachygonetria *sp.) in 33.3% to 92.5% and in turtles (European Pond Turtle and red-Eared Sliders) only in 10.0% to 16.7%. This parasite is common in herbivore reptiles. Oxyurid nematoda have developed a commensal relationship with their host [21,36,37].

**Figure 46:**
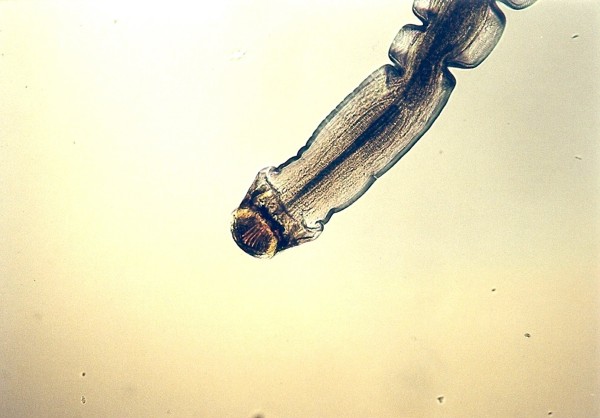
**Anterior end of *Camallanus *sp. in European Pond Tortoise (*Emys orbicularis*)**.

Strongylid nematoda (43.7%) and *Balantidium *sp. (26.2%) were found mostly in Chelonians. Among Strongylid nematoda, *Camallanus *sp. (Figure 46) were most frequently present. Ciliated protozoan *Balantidium *sp. is an important commensal organism but may reach high levels in the presence of gastrointestinal diseases [33].

**Figure 47:**
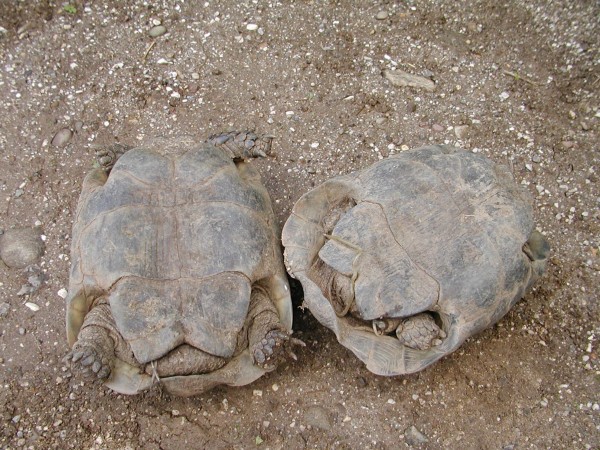
**Ascarid invasion (*Angusticaecum *sp.) in Spur-thighed Tortoise (*Testudo graeca*)**.

**Figure 48:**
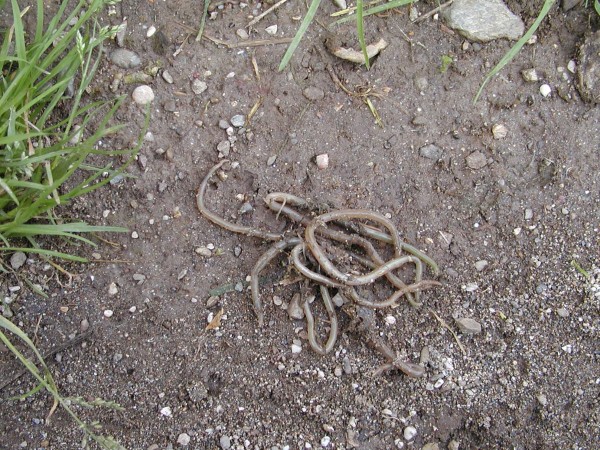
**Convolute of ascarid (*Angusticaecum *sp.) after medical treatment in Spur-thighed Tortoise (*Testudo graeca*)**.

**Figure 49:**
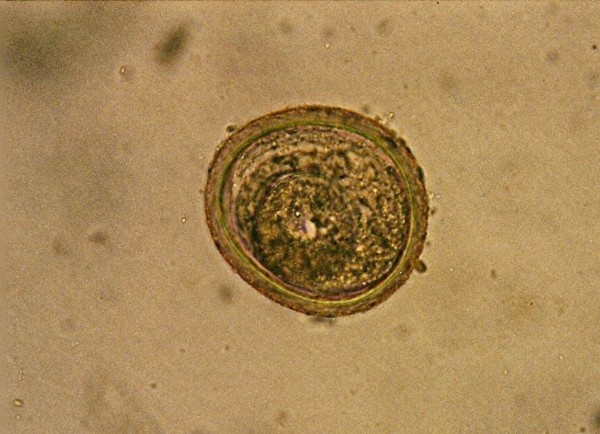
**Ascarid egg of Spur-thighed Tortoise (*Testudo graeca*)**.

Ascarid nematoda (20.3%) (Figures 47, 48 and 49) were established in Spur-thighed Tortoises in 56.9% and in Hermann's Tortoises in 11.5%. These parasites migrate through various organ systems and can lead to inflammatory lesions in the lung and other organs, which was also confirmed in our investigation. Similar findings are described by other authors. They describe secondary diarrhoea, anorexia, vomiting and loss of condition [26,37,38].

Trematoda eggs, in surprising contrast to the literature data - where this parasite is rarely mentioned in turtles - were found in high percentage in Marginated Tortoises in 60.0% and Spur-thighed Tortoises in 26.4% in our investigation. Clinical signs were not observed. Klingenberg [23] described that flukes are often seen in aquatic turtles eating fish and frogs.

In our investigation *Strongyloides *sp., Cestoda and *Nyctotherus *sp. were also detected in less than 5%. Prevalence of *Nyctotherus *sp. was lower than described by other authors [23,33].

Ectoparasites in two Indian Star Tortoises, two Radiated Tortoises and one Hermann's Tortoise were *Amblyomma *sp.

*Hyalomma *sp. ticks were found only in four Spur-thighed Tortoises. *Hyalomma aegyptium *in Spur-thighed Tortoises were also described by Tavassoli *et al*. [39]. The authors mention that only adults are specific for tortoises.

One turtle had migration of fly larvae *Calliphoridae *(*Lucilia *sp.) because of the damage on the skin caused by Hooded Crows' bites. We could not find any literature data about Crows being harmful for turtles.

## Conclusions

Many reptiles, before they even reach the pet store, die from rough handling during capture and shipping. The level of care, diet and habitat that reptiles need far outweigh that for other animals like dogs and cats, and the average person cannot adequately address these unique needs. Exotic reptile species originating directly from the wild can be carriers of many different parasites, some of which can infect humans. However, by practising good sanitation and personal hygiene, and keeping snakes, lizards and turtles out of the food preparation areas, it is possible to minimize the risk. All reptiles have to be examined and tested for specific pathogens (endo and ectoparasites, salmonella, leptospira, etc.) prior to introducing them to our homes. In our investigation we could not establish the causes of death, but we can conclude that the presence of different endo- and ectoparasites have an important role on the health status of reptiles and on the development of other diseases.

## Competing interests

The authors declare that they have no competing interests.

## Authors' contributions

AD, RLK and KV have been involved in the initial design of the study and protocols. AVR has been responsible for the parasitological work. AD has been the main responsible for data analysis in corporation with UM. All authors have contributed substantially to the editing of the manuscript.
